# Advanced Multimodality Cardiovascular Imaging of Supravalvular Aortic Stenosis in Williams-Beuren Syndrome

**DOI:** 10.1161/CIRCIMAGING.124.016733

**Published:** 2024-07-12

**Authors:** Antonino Micari, Federica Pergolizzi, Faraz Pathan, Christian Booz, Vitali Koch, Laura M. Chisari, Concetta Zito, Silvio Mazziotti, Tommaso D’Angelo

**Affiliations:** 1 DIMED Department, Cardiology Unit (A.M., C.Z.), University Hospital Messina, Italy.; 2 BIOMORF Department, Diagnostic and Interventional Radiology Unit (F. Pergolizzi, L.M.C., S.M., T.D.), University Hospital Messina, Italy.; 3 Department of Cardiology, Nepean Hospital, University of Sydney, Penrith, NSW, Australia (F. Pathan).; 4 Division of Experimental Imaging, Department of Diagnostic and Interventional Radiology, University Hospital Frankfurt, Frankfurt am Main, Germany (C.B., V.K.).; 5 Department of Radiology and Nuclear Medicine, Erasmus MC, Rotterdam, the Netherlands (T.D.).

**Keywords:** aortic valve stenosis, computed tomography angiography, forehead, magnetic resonance imaging, pulmonary artery

A 37-year-old man, who had intellectual disability since birth, underwent cardiac magnetic resonance imaging (MRI) to further evaluate suspected aortic valve dysplasia detected in a previous echocardiogram.

The physical examination highlighted a broad forehead, wide mouth with full lips, low and flat nasal bridge, epicanthus, short stature, and early graying of hair. The patient also had concurrent hypogonadotropic hypogonadism, somatotropic hormone deficiency, and visual disturbances (ie, divergent strabismus). Past medical history included diverticulitis and inguinal hernioplasty surgery.

Despite undergoing several cardiological examinations as part of endocrinologic follow-up, the patient had never reported specific cardiac symptoms. Previous transthoracic echocardiograms showed normal left ventricular systolic function and moderate aortic stenosis, attributed to a bicuspid aortic valve.

A cardiovascular examination revealed a systolic aortic murmur (grade 3/6 on the Levine scale). The latest transthoracic echocardiogram indicated a peak transvalvular aortic velocity of 3.2 m/s and a mean gradient of 25 mm Hg on continuous wave Doppler, with mild aortic regurgitation. However, due to poor acoustic window and uncertainty in valve anatomy assessment, suspicion of possible supravalvular stenosis arose. Transesophageal echocardiography was not performed due to patient anatomy and anesthesia risks, leading to a referral for cardiac MRI.

Cardiac MRI was performed using a short protocol comprising solely balanced steady-state free-precession sequences for cine imaging and phase-contrast sequences for flow imaging due to the patient’s limited cooperation.

Cine imaging showed the presence of a thin and mobile membrane at the sinus-tubular junction with a doming effect in the systole that was responsible for supravalvular aortic stenosis (SVAS; Figure [A]; Video S1). Other cardiovascular abnormalities found in cine sequences were mild aortic regurgitation (Video S2) and mild supravalvular pulmonary stenosis.

**Figure.**
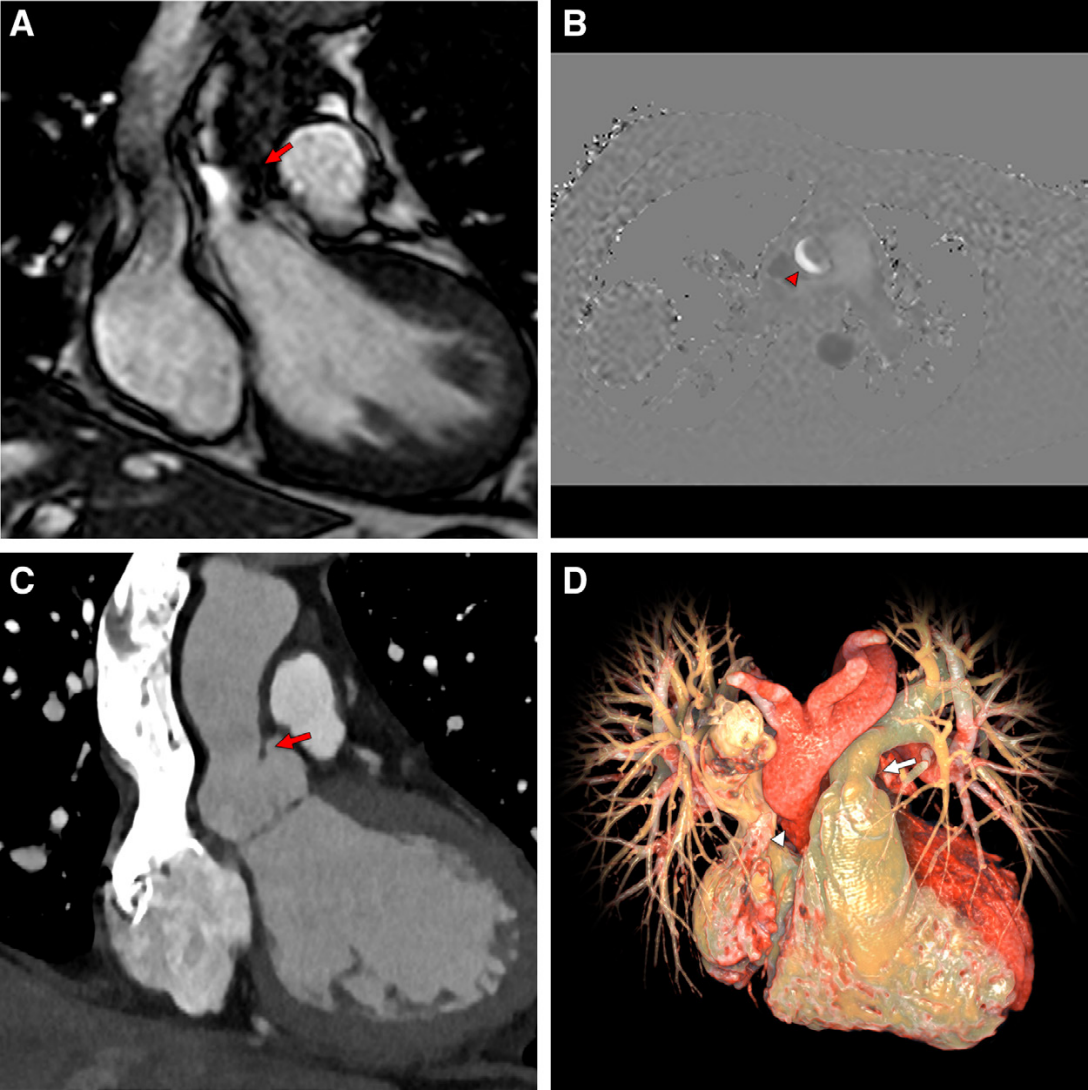
**Multimodality imaging assessment of supravalvular aortic stenosis in a 37-year-old man. A**, Cardiac magnetic resonance imaging (MRI) balanced steady-state free-precession sequence along the left ventricular outflow tract, showing turbulent blood flow above the aortic valve during systole (red arrow). **B**, Cardiac MRI through-plane phase-contrast velocity mapping obtained at the sinotubular junction level shows a crescent-like appearance of the aortic flow (red arrowhead). **C**, Cardiac computed tomography angiography (CTA) multiplanar reconstruction along the left ventricular outflow tract confirms the presence of a supravalvular aortic membrane (red arrow), with hourglass stenosis of the proximal ascending aorta. **D**, Cardiac CTA photorealistic volume rendering image showing the hourglass appearance of the proximal ascending aorta (white arrowhead) and the mild supravalvular pulmonary stenosis (white arrow).

Flow imaging demonstrated a crescent-like appearance of the blood flow at the level of the SVAS (Figure [B]; Video S3).

To better assess the sizing of the supravalvular aortic lumen and to explore potential additional congenital cardiothoracic anomalies, the patient underwent cardiac computed tomography angiography, with prospective ECG gating to minimize the radiation exposure. The examination confirmed the narrowing at the level of the sinus-tubular junction (with an hourglass appearance of the proximal section of the ascending aorta) and the presence of a membrane between the sinus-tubular junction and the left coronary cusp configuring the SVAS (Figure [C]). The mild supravalvular stenosis of the pulmonary artery observed at the previous cardiac MRI examination was also confirmed (Figure [D]). No other cardiothoracic anomalies were demonstrated.

Based on the patient’s medical history and imaging findings, the diagnostic suspicion of Williams-Beuren syndrome was raised.

The patient was referred for genetic evaluation, and the diagnosis was confirmed using an array-based comparative genomic hybridization technique, which showed a microdeletion involving the 7q11.23 region, known to be associated with the Williams-Beuren syndrome.

The Williams-Beuren syndrome is a rare congenital disorder with the involvement of the skeletal, nervous, and cardiovascular systems. Cardiovascular defects are the main cause of death in these patients, and their pathogenesis primarily depends on the defect of elastin, normally present in the wall of arterial vessels. Specifically, elastic fibers are composed of polymers of elastin and allow the artery to respond to the hemodynamic stress, avoiding deformation or dilation of the vessel. Patients with William’s syndrome have a reduced amount of elastin in the wall of the arterial vessel, resulting in increased vessel stiffness.^1^ Moreover, using a murine model of elastin-lacking vascular smooth muscle cells, Karnik et al^2^ showed that elastin also contributes to the modulation of the proliferation and migration of vascular muscle cells. Therefore, a qualitative or quantitative reduction of elastin can cause hypertrophy of the arterial vessel wall.^2^

Among all the cardiovascular defects associated with the syndrome, SVAS and supravalvular pulmonary stenosis are the most common findings.^3^

Anatomically, SVAS can be classified into 3 subtypes: (1) a stenosis caused by a supravalvular membrane, as in the clinical case presented; (2) an hourglass stenosis, with a ring-shaped narrowing in the proximal portion of the ascending aorta; and (3) a stenosis secondary to diffuse hypoplasia of the ascending aorta.^4^

Although SVAS in patients with William’s syndrome has widely been described in scientific literature, to the best of our knowledge, no reports of multimodality imaging with magnetic resonance flow imaging and high-resolution cardiac computed tomography angiography have ever been published.

Moreover, our images show a hybrid subtype of SVAS because the supravalvular membrane coexists with an hourglass stenosis of the proximal ascending aorta, immediately above the sinus-tubular junction (Figure [C]). This complex anatomic anomaly results in the appearance of a crescent-like aspect of blood flow on magnetic resonance flow-imaging sequences (Figure [B]).

Our clinical case highlights the importance of advanced multimodal imaging in the study of rare valve anomalies. High spatial resolution imaging modalities, such as MRI and computed tomography, are fundamental for the correct anatomic diagnosis in the suspicion of valve disease, in which the echocardiographic examination alone might be inconclusive.

## ARTICLE INFORMATION

### Acknowledgments

The authors acknowledge Dr Clotilde Buceti for her assistance in providing detailed clinical patient’s data.

### Sources of Funding

None.

### Disclosures

None.

### Supplemental Material

Videos S1–S3

## Supplementary Material
