# Supplementary material for: Advanced Multimodality Cardiovascular Imaging of Supravalvular Aortic Stenosis in Williams-Beuren Syndrome
Source: Circ Cardiovasc Imaging. 2024 Jul 12;17(11):e016733. doi: 10.1161/CIRCIMAGING.124.016733 (PMC11575900; doi:10.1161/CIRCIMAGING.124.016733)
Supplement: Supplementary file 1 [file hci-17-e016733-s001.docx]

## **SUPPLEMENTAL MATERIAL**

**Video S1.** Cardiac-MRI bSSFP sequence along the left ventricular outflow tract showing turbulent blood flow above the aortic valve, due to the supravalvular stenosis.

**Video S2**. Cardiac-MRI bSSFP sequence on a parasternal long-axis view showing mild aortic insufficiency.

**Video S3.** Cardiac-MRI through-plane phase-contrast velocity mapping obtained at the sinotubular junction level, shows a “crescent-like appearance” of the aortic accelerated flow. Velocity encoding was set to 3 m/s.
